# Endoscopic submucosal dissection of a large cavernous hemangioma in the proximal esophagus

**DOI:** 10.1055/a-2707-3514

**Published:** 2025-10-02

**Authors:** Andreas Probst, Tina Schaller, Tobias Weber, Helmut Messmann, Markus Wolfgang Scheppach

**Affiliations:** 1 39694 Department of Gastroenterology, University Hospital Augsburg, Augsburg, Germany; 2 39694 Institute of Pathology and Molecular Diagnostics, University Hospital Augsburg, Augsburg, Germany

Esophageal subepithelial tumors (SETs) are often diagnosed incidentally and the treatment strategy can be challenging. In asymptomatic lesions without proven histology, endoscopic surveillance is justified 1 . However, the uncertainty regarding the risk of progression and the need for frequent surveillance endoscopies have to be balanced against the possibility and the potential risks associated with minimally invasive endoscopic resection.

A 42-year-old woman without preexisting comorbidities underwent upper gastrointestinal endoscopy because of recurrent epigastric pain. The patient was otherwise asymptomatic. In the proximal esophagus, a nodular SET with a bluish-purple appearance and a diameter of about 20 mm was seen ( Fig. 1 ). The lesion was located 1 cm distal to the upper esophageal sphincter. Magnetic resonance imaging confirmed a lumen-occupying mass in the proximal esophagus, 27 × 20 × 14 mm in diameter. Endoscopic ultrasound showed an inhomogeneous hypoechoic and well-demarcated mass in the submucosal layer ( Fig. 2 ). According to the lesion’s morphology, cavernous hemangioma was suspected. Biopsies were not taken because of the bleeding risk and the exact diagnosis of the asymptomatic mass remained unclear after diagnostic workup.

**Fig. 1:**
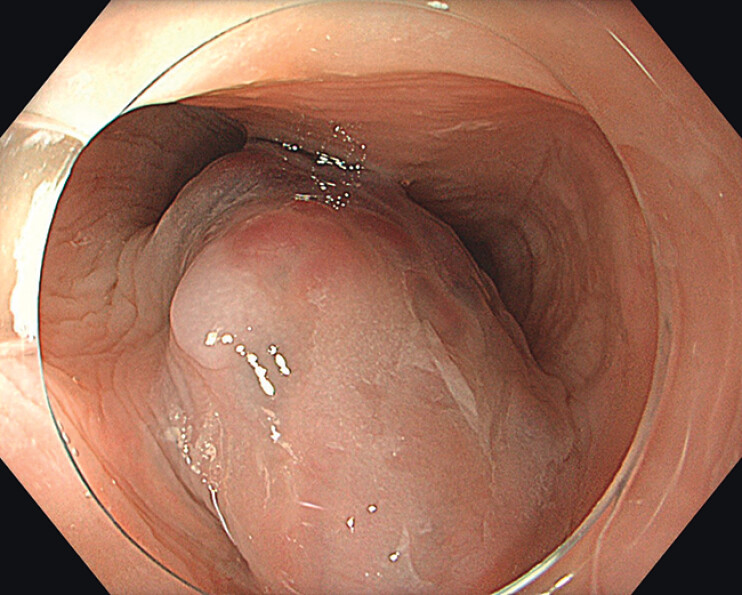
Endoscopic appearance of the subepithelial tumor in the proximal esophagus.

**Fig. 2:**
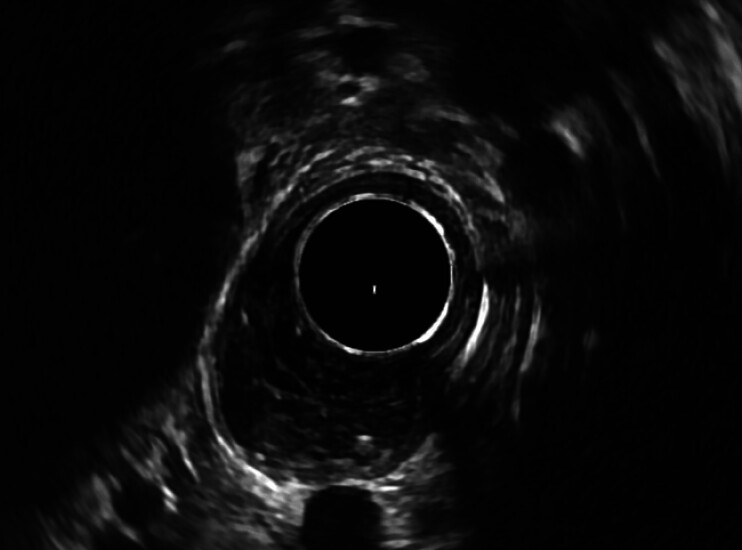
Endoscopic ultrasound confirmed an inhomogeneous hypoechoic and well-demarcated mass localized in the submucosa.

An endoscopic surveillance strategy versus endoscopic resection to confirm an exact diagnosis and avoid follow-up examinations were discussed. The patient opted for endoscopic resection and endoscopic submucosal dissection (ESD) was performed under general anesthesia ( Video 1 ). En bloc resection was possible without any bleeding or other complications ( Fig. 3 ). The resection specimen showed a multinodular, purple mass 2 cm in diameter and located in the submucosal layer ( Fig. 4 ). The further course was uneventful. Histopathology confirmed a submucosal cavernous hemangioma, which was resected R0 ( Fig. 5 ).

Endoscopic submucosal dissection of a large cavernous hemangioma located in the proximal esophagus.Video 1

**Fig. 3:**
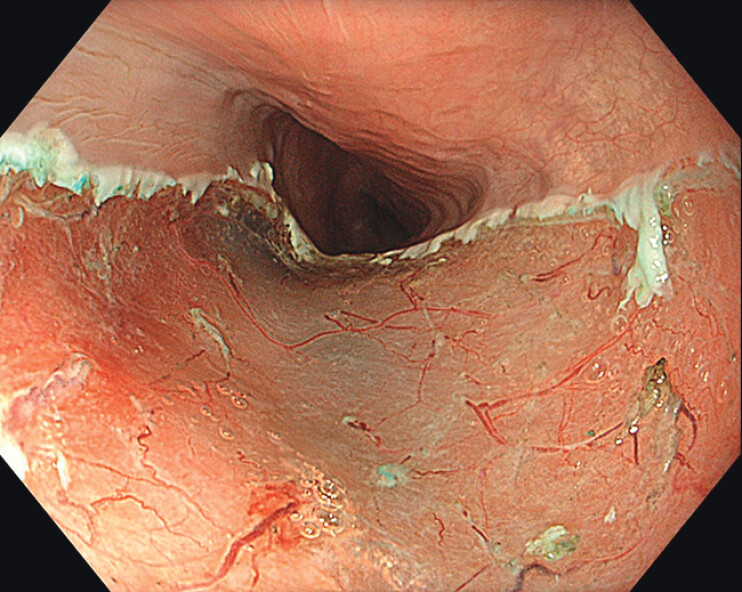
Resection area after endoscopic submucosal dissection in the proximal esophagus.

**Fig. 4:**
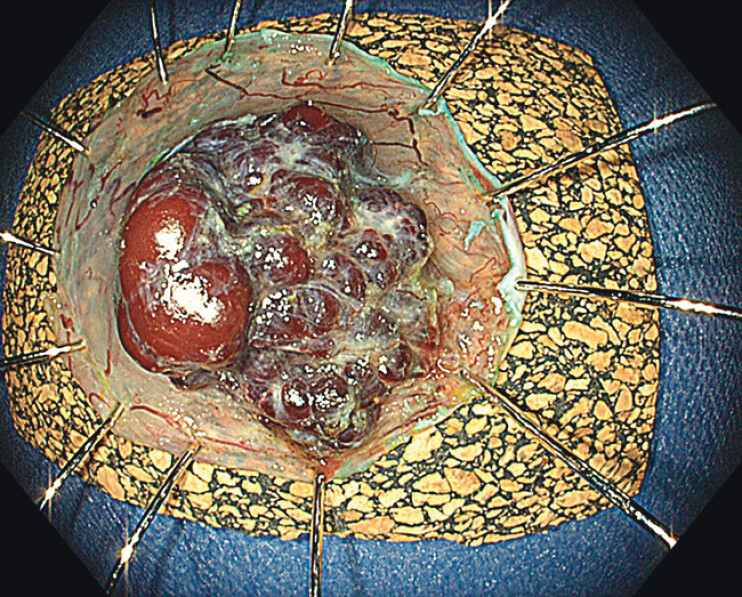
The resection specimen after endoscopic submucosal dissection, showing a multinodular purple mass in the submucosa (specimen was fixed onto cork with needles; view from the submucosal direction).

**Fig. 5:**
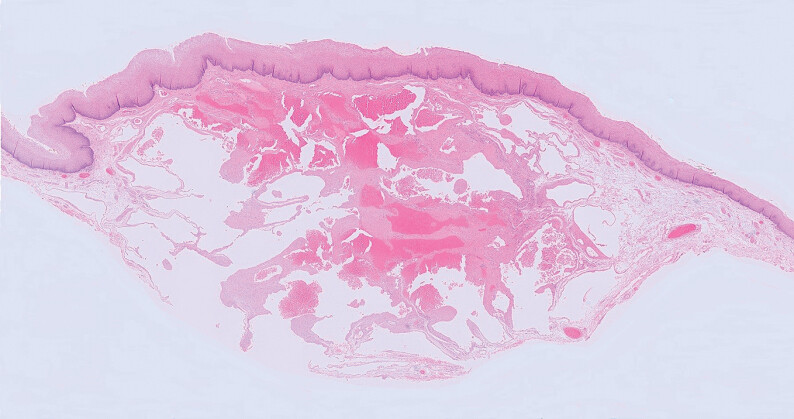
Histopathology showed a vascular neoplasia with large cystically dilated vessels (partially filled with blood) and focally fibrotic walls. The lesion was localized in the submucosal layer and covered by normal squamous epithelium (hematoxylin and eosin).

The diagnosis of esophageal cavernous hemangioma can be suspected macroscopically but usually cannot be confirmed without resection of the lesion 2 3 4 5 . When performed in experienced hands, ESD offers a minimally invasive and safe treatment option that confirms the diagnosis and achieves a definitive treatment.

Endoscopy_UCTN_Code_CCL_1AB_2AC

## Notes

Endoscopy E-Videos https://eref.thieme.de/e-videos
*E-Videos* is an open access online section of the journal *Endoscopy* , reporting on interesting cases and new techniques in gastroenterological endoscopy. All papers include a high-quality video and are published with a Creative Commons CC-BY license. Endoscopy E-Videos qualify for HINARI discounts and waivers and eligibility is automatically checked during the submission process. We grant 100% waivers to articles whose corresponding authors are based in Group A countries and 50% waivers to those who are based in Group B countries as classified by Research4Life (see: https://www.research4life.org/access/eligibility/ ). This section has its own submission website at https://mc.manuscriptcentral.com/e-videos .
